# Human Adenovirus Type 37 Uses α_V_β_1_ and α_3_β_1_ Integrins for Infection of Human Corneal Cells

**DOI:** 10.1128/JVI.02019-16

**Published:** 2017-02-14

**Authors:** Rickard J. Storm, B. David Persson, Lars Nygård Skalman, Lars Frängsmyr, Mona Lindström, Greg Rankin, Richard Lundmark, Fatima Pedrosa Domellöf, Niklas Arnberg

**Affiliations:** a Division of Virology, Department of Clinical Microbiology, Umeå University, Umeå, Sweden; b Department of Medical Biochemistry and Biophysics, Umeå University, Umeå, Sweden; c Department of Clinical Sciences, Ophthalmology, Umeå University, Umeå, Sweden; d Department of Integrative Medical Biology, Umeå University, Umeå, Sweden; e Department of Public Health and Clinical Medicine, Norrland University Hospital (NUS), Umeå University, Umeå, Sweden; International Centre for Genetic Engineering and Biotechnology

**Keywords:** adenoviruses, cornea, epidemic keratoconjunctivitis, integrins

## Abstract

Epidemic keratoconjunctivitis (EKC) is a severe, contagious ocular disease that affects 20 to 40 million individuals worldwide every year. EKC is mainly caused by six types of human adenovirus (HAdV): HAdV-8, -19, -37, -53, -54, and -56. Of these, HAdV-8, -19, and -37 use sialic acid-containing glycans as cellular receptors. αVβ3, αVβ5, and a few additional integrins facilitate entry and endosomal release of other HAdVs. With the exception of a few biochemical analyses indicating that HAdV-37 can interact physically with αVβ5, little is known about the integrins used by EKC-causing HAdVs. Here, we investigated the overall integrin expression on human corneal cells and found expression of α2, α3, α6, αV, β1, and β4 subunits in human corneal *in situ* epithelium and/or in a human corneal epithelial (HCE) cell line but no or less accessible expression of α4, α5, β3, or β5. We also identified the integrins used by HAdV-37 through a series of binding and infection competition experiments and different biochemical approaches. Together, our data suggest that HAdV-37 uses αVβ1 and α3β1 integrins for infection of human corneal epithelial cells. Furthermore, to confirm the relevance of these integrins in the HAdV-37 life cycle, we developed a corneal multilayer tissue system and found that HAdV-37 infection correlated well with the patterns of αV, α3, and β1 integrin expression. These results provide further insight into the tropism and pathogenesis of EKC-causing HAdVs and may be of importance for future development of new antiviral drugs.

**IMPORTANCE** Keratitis is a hallmark of EKC, which is caused by six HAdV types (HAdV-8, -19, -37, -53, -54, and -56). HAdV-37 and some other HAdV types interact with integrin αVβ5 in order to enter nonocular human cells. In this study, we found that αVβ5 is not expressed on human corneal epithelial cells, thus proposing other host factors mediate corneal infection. Here, we first characterized integrin expression patterns on corneal tissue and corneal cells. Among the integrins identified, competition binding and infection experiments and biochemical assays pointed out αVβ1 and α3β1 to be of importance for HAdV-37 infection of corneal tissue. In the absence of a good animal model for EKC-causing HAdVs, we also developed an *in vitro* system with multilayer HCE cells and confirmed the relevance of the suggested integrins during HAdV-37 infection.

## INTRODUCTION

Human adenoviruses (HAdVs) are common human pathogens that variously cause infections in the intestine, the airways, the urinary tract, the tonsils, and the eyes (1). Currently, more than 60 HAdV types have been identified, which have been classified further into seven species (A to G). Epidemic keratoconjunctivitis (EKC), a severe and contagious ocular disease, is mainly caused by six members of HAdV species D (HAdV-8, -19, -37, -53, -54, and -56). EKC is characterized by pain, tearing, edema, and reduced vision that may last for months or years. Even though many other HAdVs of species B, C, and E in particular can also infect the eye, a hallmark of EKC is strong involvement of the cornea (2, 3). It is estimated that worldwide, EKC affects 20 to 40 million individuals annually. The disease is considered to be endemic to Japan and is associated with an enormous socioeconomic cost (4, 5). Currently, no treatment is available for EKC.

Several molecules have been identified as cellular receptors for HAdVs, including the coxsackie and adenovirus receptor (CAR), CD46, and desmoglein-2 (6–8). EKC-causing HAdVs interact physically with CAR (9) and use CD46 as a receptor on Chang C cells (10). On corneal cells, EKC-causing HAdVs use sialic acid-containing glycoproteins with motifs that correspond to the glycan present on GD1a ganglioside (11). After initial attachment to the cell, which is mediated by the knob domain of the viral fiber protein irrespective of the cellular receptor used, most HAdVs engage cellular integrins, in particular αVβ3 and αVβ5, as coreceptors for entry into host cells and endosomal release (12–15). HAdV-5 also uses integrins αVβ1 and α3β1 as coreceptors on certain cell types (16, 17). Integrins are a large family of membrane-bound, heterodimeric glycoproteins consisting of an α and a β subunit (18–20). In humans, 18 α and 8 β subunits have been identified that form 24 unique heterodimeric complexes. Upon binding to ligands, integrins transfer both so-called inside-out signals and outside-in signals. These signals regulate a number of processes, such as cell migration, endocytosis, and immune responses. Extracellular ligands interact with integrins, including αV-containing integrins, through conserved ligand motifs such as the Arg-Gly-Asp (RGD) motif (19). Examples of RGD-containing ligands are vitronectin, fibronectin, and laminin 511, which are all known to interact with a range of different integrin α subunits (18, 21). Most HAdV-integrin interactions are mediated by a conserved RGD motif located in a protruding loop of the penton base protein (12), which is shared by all HAdVs except the two members of HAdV species F (HAdV-40 and HAdV-41) (22).

Previously published enzyme-linked immunosorbent assay (ELISA)-based results suggested that HAdV-37 virions interact more efficiently with integrin αVβ5 than other HAdVs (23). Unfortunately, as most integrin expression studies of the cornea have been performed in rats or mice (24–26), the expression pattern of αVβ5 in human corneal epithelium remains unknown. A recent proteomic analysis of human corneal tissue failed to detect any expression of αVβ5 (27), suggesting that interactions with this integrin are unlikely to account for the corneal tropism of the EKC-causing HAdVs. To obtain better insight into the life cycle of EKC-causing HAdVs and to identify novel targets for antiviral drugs, we set out to identify the integrins used by these viruses to infect corneal cells.

## RESULTS

### β3 and β5 integrin subunits are absent from superficial human corneal epithelial cells.

**FIG 1:**
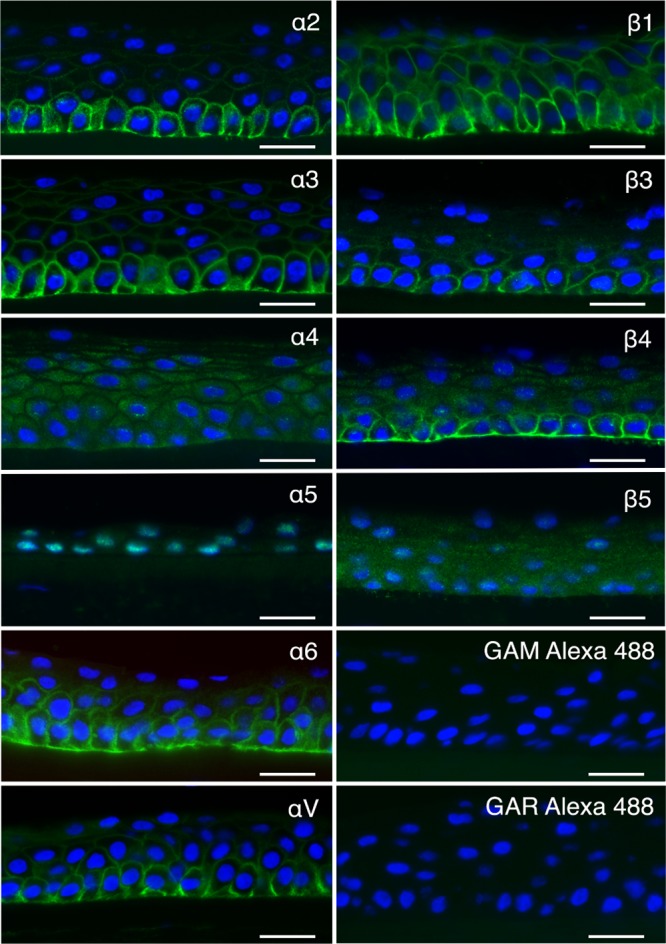
Cross sections of human corneas labeled (in green) with antibodies against integrin α2 (P1E6), α3 (ASC-1), α4 (P4C2), α5 (P1D6), α6 (MP4F10), αV (272-17E6), β1 (P5D2), β3 (MHF4), β4 (422325), and β5 (H00051706-D01P), as well as secondary-antibody-only controls GAM Alexa 488 and GAR Alexa 488. Notice the absence of specific labeling of the cell surface with antibodies against integrin α4, α5, and β5. Bar, 25 μm. Nuclei are stained using DAPI (blue). Representative images from 3 cornea explants are shown.

**FIG 2:**
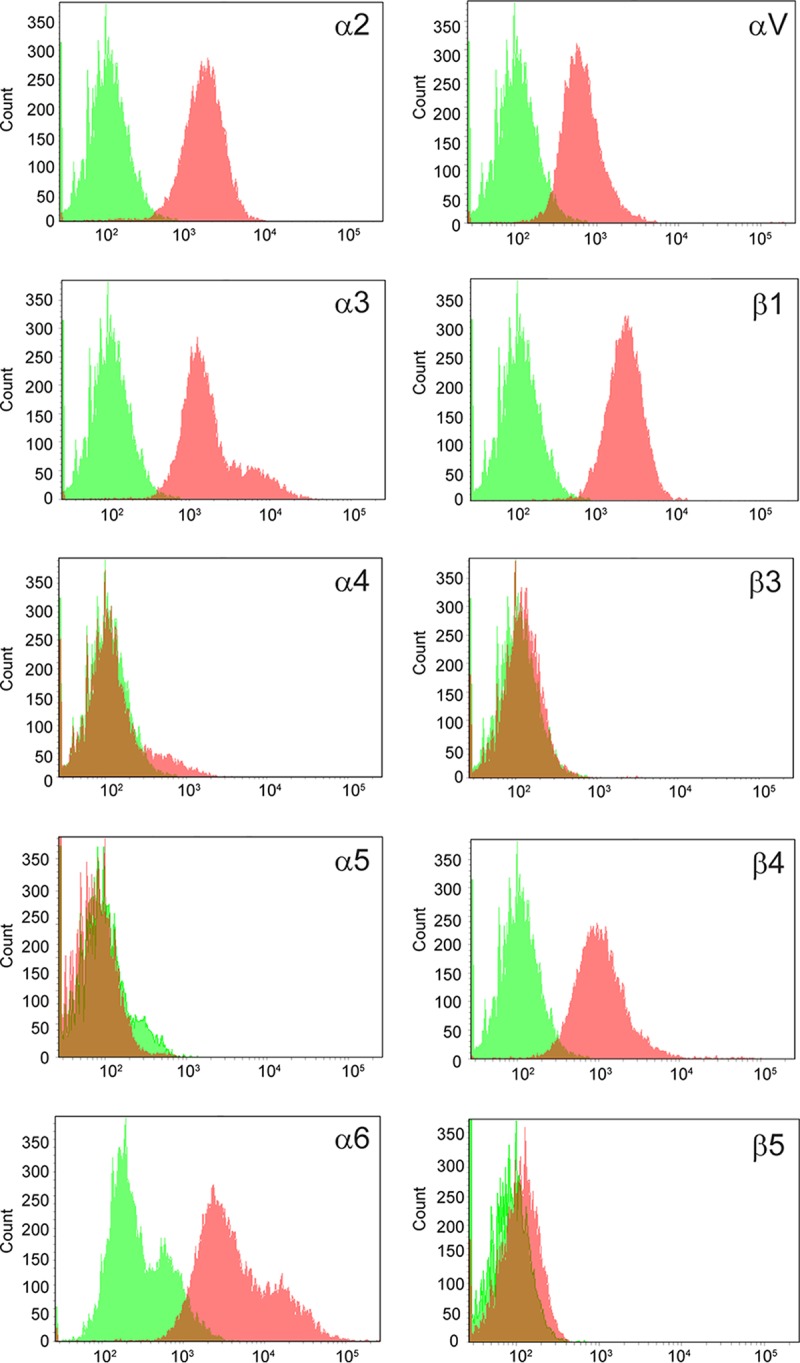
Flow cytometry analysis of integrin subunit expression on HCE cells. The following antibodies were used to detect respective integrin subunits: α2 (P1E6), α3 (P1B5), α4 (P4C2), α5 (P1D6), α6 (GoH3), αV (272-17E6), β1 (P5D2), β3 (MF4), β4 (422325), and β5 (H00003693-D01P). Red and green colors represent staining in the presence and absence of primary antibody, respectively. *n* = 2.

Integrin expression in the human cornea has not been very well characterized, and the current view is actually based on extrapolations from integrin expression in mice and rats (24–26). However, a proteomic study of the human cornea indicated that there is expression of α2, α3, α6, αV, β1, and β4 integrin subunits but not any others (27). This encouraged us to investigate corneal integrin expression further in order to gain a better understanding of the tropism of EKC-causing HAdVs. To verify these findings, we started with immunohistochemical analysis of human corneal tissue sections, which confirmed the presence of α2, α3, α6, αV, β1, and β4 (Fig. 1). The staining for β4 was present mainly in lower cell layers, whereas it was found in all layers for the remaining antibodies. β1 in particular was abundantly stained throughout the cornea. The monoclonal antibody (MAb) against β3 integrin labeled the contours of the cells in the lower layers weakly, and no clear labeling could be seen in the upper layers. Sections of human corneal tissue were considered negative if there was intracellular staining and no clear staining of plasma membranes (i.e., staining with antibodies against α4, α5, and β5). Furthermore, using flow cytometry we could confirm that like corneal tissue, human corneal epithelial (HCE) cell surfaces also express α2, α3, α6, αV, β1, and β4 subunits (Fig. 2). No or very weak staining of α4, α5, β3, and β5 subunits was observed. The functioning of anti-α4, -α5, -β3, and -β5 antibodies was validated using α5, β3-, and β5-positive A549 cells and α4-positive MOLT-16 cells (data not shown). HCE cells have been used extensively in the past to study receptors used by EKC-causing HAdVs. Based on these results, we conclude that subunits α2, α3, α6, αV, β1, and β4 are accessible in the human corneal epithelium *in vivo* and *in vitro*. We also found that the β3 subunit is less accessible and that the β5 subunit is completely absent from human corneal cells. This suggests that these two integrin subunits are of less or no relevance to HAdV-37 infection of HCE cells. Given the resemblance in integrin expression patterns between these cells and primary human corneal tissue, we also concluded that these cells would be a useful model system to study integrin usage by EKC-causing HAdVs.

### Antibodies to α3, αV, and β1 inhibit HAdV-37 infection of corneal epithelial cells.

**FIG 3:**
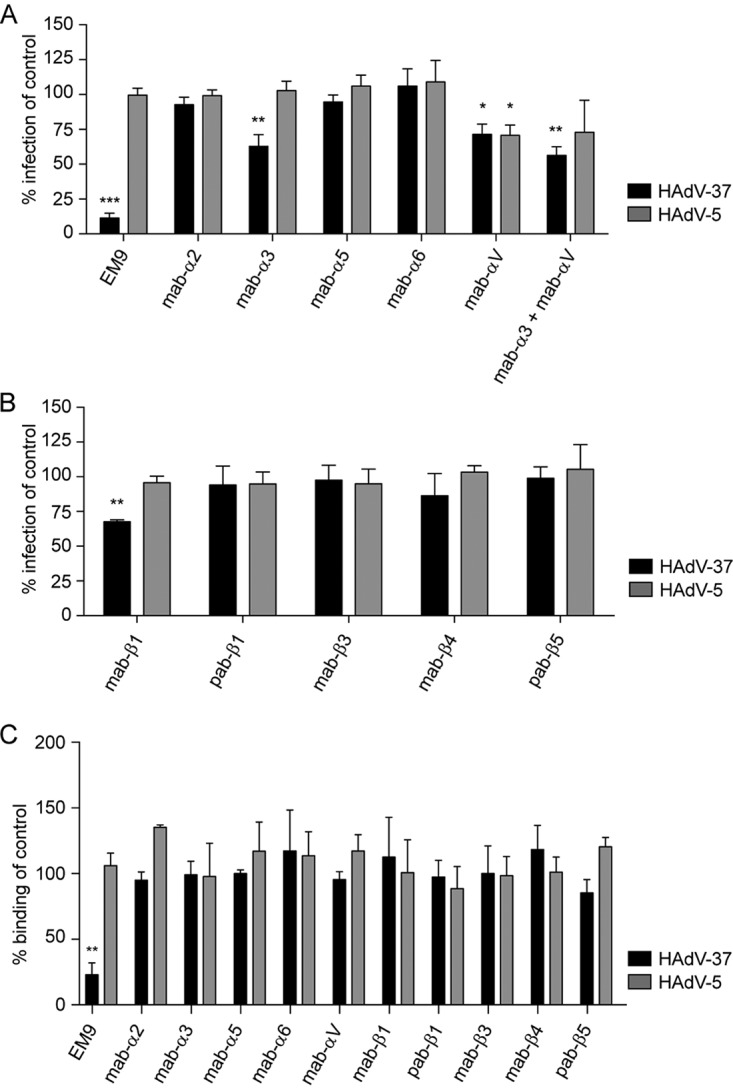
Effects of preincubating HCE cells with anti-integrin antibodies on HAdV-37 and HAdV-5 infection and binding. (A) Effect of antibodies against integrin α subunits on HAdV-37 and HAdV-5 infection. (B) Effect of antibodies against integrin β subunits on HAdV-37 and HAdV-5 infection. (C) Effect of antibodies against integrin α and β subunits on HAdV-37 and HAdV-5 binding. Infection was assayed by counting virus-positive cells by immunofluorescence, and binding was assayed by quantitating ^35^S-labeled virus association with cells. The results are presented as percentages of the control (i.e., untreated cells). The following antibodies were used: α2 (P1E6), α3 (P1B5), α4 (P4C2), α5 (P1D6), α6 (GoH3), αV (272-17E6), β1 (P5D2), β3 (MHF4), β4 (422325), and β5 (H00003693-D01P). The anti-GD1a specific MAb EM9 was used as a positive control. Results are shown as a percentage of the value for the control. *, *P* < 0.05; **, *P* < 0.01; ***, *P* < 0.001. *n* = 3.

To identify the relative importance of specific α and β subunits during infection of EKC-causing HAdV, we next preincubated HCE cells with subunit-specific MAbs or polyclonal antibodies (PAbs) prior to infection. These antibodies were selected on the basis of their potential to interfere with integrin function and ligand interaction. Antibodies specifically recognizing α3 or αV both inhibited HAdV-37 infection by approximately 30% (Fig. 3A). Pretreatment of cells with antibodies to both α3 and αV had an additive effect, further increasing the inhibition to 50%. As a control, we used HAdV-5, which has previously been reported to use several α subunits, including α3 and αV, as coreceptors on various cell lines. Of all the antibodies tested, only the MAb-αV antibody inhibited HAdV-5 infection of HCE cells by approximately 30%. To better understand the relative importance of the β subunits during HAdV-37 infection of HCE cells, antibodies specifically recognizing several β subunits were also tested. We found that only one antibody, directed against the β1 subunit, had any effect on HAdV-37 infection, reducing the infection by 30%. None of the β subunit-specific antibodies inhibited HAdV-5 infection (Fig. 3B). In addition, none of the antibodies affected virion binding to HCE cells (Fig. 3C), suggesting that α3, αV, and β1 subunits are important for HAdV-37 infection of, but not for attachment to, human corneal epithelial cells. The anti-GD1a MAb EM9 was used as a positive control for HAdV-37 (11), as expected, and inhibited binding and infection of HAdV-37 but not of HAdV-5.

### Functional validation of integrins as coreceptors for HAdV-37.

**FIG 4:**
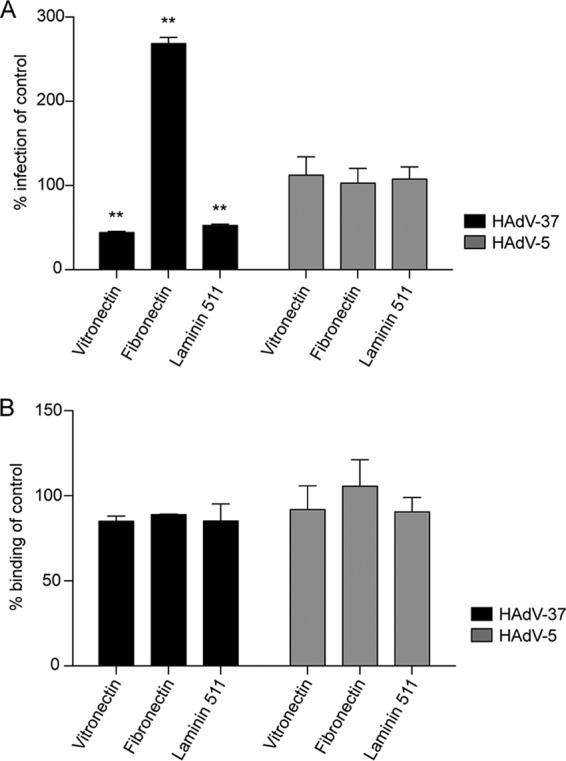
Effect of (RGD-containing) integrin-interacting ligands on HAdV infection (A) and binding (B) of HCE cells. HCE cells were pretreated with vitronectin (1 μM), fibronectin (1 μM), and laminin 511 (0.12 μM) prior to infection of HCE cells. The results are presented as a percentage of the value for the control (i.e., untreated cells). *, *P* < 0.05; **, *P* < 0.01. *n* = 3.

**FIG 5:**
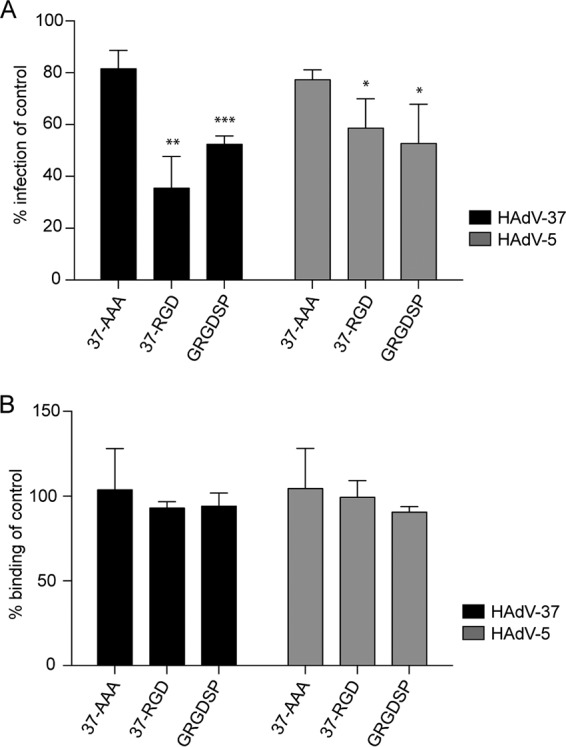
Effect of penton base-derived, RGD-containing peptides (4 mM) on HAdV-5 and HAdV-37 infection of (A) and binding to (B) HCE cells. 37-RGD, peptide mimicking RGD-containing loop of HAdV-37 penton base. 37-AAA, RGD-containing loop in HAdV-37 penton base peptide replaced with AAA. GRGDSP, peptide used previously (12) to inhibit HAdV infection of human cells. The results are presented as a percentage of the value for the control (i.e., untreated cells). *, *P* < 0.05; **, *P* < 0.01; ***, *P* < 0.001. *n* = 3.

To further investigate the importance of integrins as coreceptors for EKC-causing HAdVs, we preincubated HCE cells with natural, RGD-containing protein ligands prior to binding and infection. Vitronectin and laminin 511 inhibited HAdV-37 infection by approximately 65% and 55%, respectively (Fig. 4A). Fibronectin, on the other hand, enhanced infection almost 3-fold. HAdV-5 infection of, or binding to, HCE cells was completely unaffected by the integrin ligands. None of the ligands inhibited binding of HAdV-37 to HCE cells (Fig. 4B). We next preincubated HCE cells with a 16-residue-long peptide (37-RGD) that mimicked the exposed RGD-containing loop of the HAdV-37 penton base. In addition, to specifically examine the role of the RGD motif, two additional peptides were included, one where the RGD motif was exchanged for triple Ala (AAA; 37-AAA) and one very short peptide (GRGDSP) that inhibits HAdV infection of human M21-4 melanoma cells (12). The RGD-containing peptides inhibited HAdV-37 infection efficiently (by 50 to 65%), whereas the control peptide (37-AAA) had no effect or only a small effect (less than 20%) (Fig. 5A). Both RGD-containing peptides inhibited HAdV-5 infection of HCE cells by approximately 45%. None of the peptides affected the binding of HAdV-37 or HAdV-5 to HCE cells (Fig. 5B). Together, these results suggest that the RGD motif in the HAdV-37 penton base is of importance for infection of HCE cells.

### Colocalization of HAdV-37 with α3, αV, and β1 integrins in HCE single cells and in multilayers.

**FIG 6:**
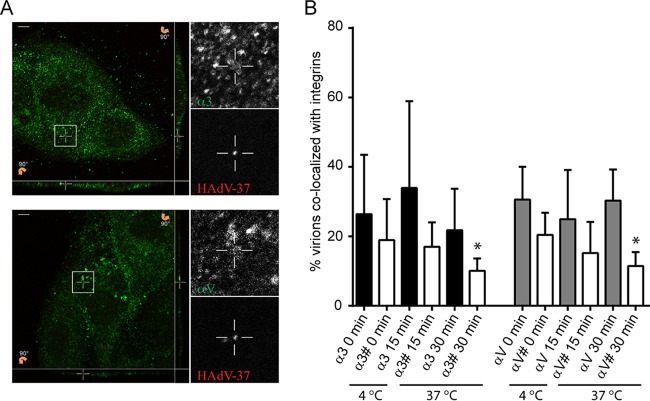
HAdV-37 virions colocalize with α3 and αV integrins on HCE cells. Alexa Fluor 555-labeled HAdV-37 virions (in orange) were incubated with HCE cells at 37°C for different time points and subsequently stained for α3 (P1B5) or αV (272-17E6) integrins (in green). (A) Representative confocal images of HAdV-37 virions colocalizing with integrin antibodies after 15 min (top, α3; bottom, αV). Slices 90° tilted show optical sections of the *x-z* and *y-z* planes at the bottom and right side, respectively, of each image on the left. The marked squares display a colocalization event, enlarged to the right in the center of the crosshairs in each box and in the *x-y*, *x-z*, and *y-z* sections. Scale bars, 5 μm. (B) Quantification of the percentage of colocalized HAdV-37 virions with α3 and αV integrins at different time points in HCE cells. Data were collected from 5 to 7 z stacks with at least 2 cells per stack. #, Bars represent data from a pixel shift analysis to test if colocalization occurs by chance. *, *P* < 0.05. *n* = 1.

To gain further insight into the interaction between HAdV-37 and integrins on a cellular level, we quantified costaining of AF555-labeled HAdV-37 virions and integrins αV and α3 at various time points after infection. In order to fully examine the entry process, we incubated virions with cells on ice for 30 min (*T* = 0) or at 37°C for 15 or 30 min (*T* = 15 or *T* = 30), washed away nonbound virions, and analyzed virion colocalization with integrins. At these time points, approximately 20 to 40% of all labeled virions colocalized with either α3 or αV subunits on HCE cells (Fig. 6A and B). To validate the colocalization, we performed a pixel shift analysis were the pixels in the red HAdV-37-AF555 stain was shifted 1 μm in *x* and *y* directions as well as 0.5 μm in *z* direction before analysis. The percentage of colocalization between the shifted HAdV-37 and original HAdV-37 and integrins was compared using a paired *t* test (*P* = 0.05). The results showed a statistically significant difference between true and random colocalization in the 30-min samples for αV (*P* = 0.0119) and α3 (*P* = 0.0447) and for HAdV-37.

**FIG 7:**
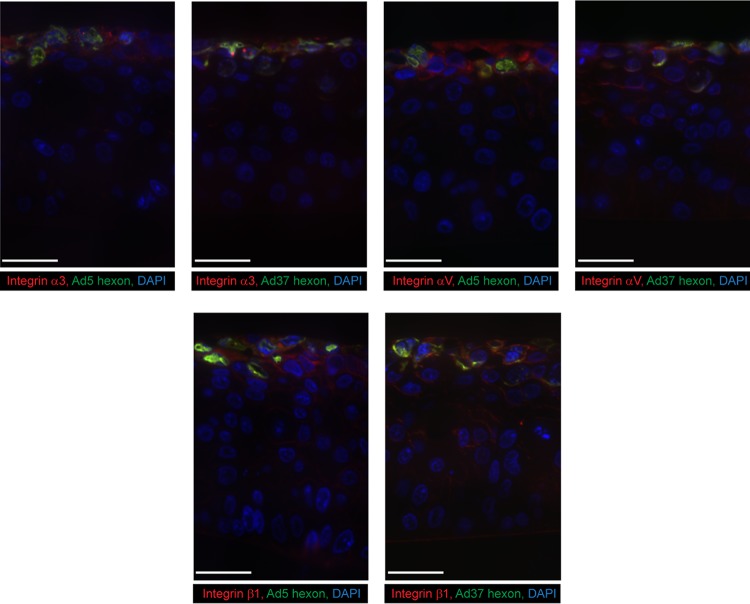
HAdV infection (in green) and expression of integrin subunits α3, αV, and β1 (in red) on HCE cells grown as multilayers at the air-liquid interphase. HAdV-5 and -37 were infected from the apical (upper) side. Antibodies used for staining were P1B5 (α3), 272-17E6 (αV), P5D2 (β1), and B025/AD51 (adenovirus hexon). Nuclear stain is shown in blue. Staining with secondary antibodies only was completely negative. White bar, 50 μm. *n* = 2.

HCE cells can polarize and develop to a multilayer when grown on an air-liquid interface (ALI) (28). To investigate integrin expression in this system and to examine integrin function in HAdV infection, we generated these cultures and infected them with HAdV-37 and HAdV-5. The HCE cells were grown for 10 days, which generated a multilayer (6 to 9 layers) similar to the *in vivo* situation with five to seven layers of corneal epithelial cells (29). A total of 2 × 10^5^ HAdV-37 or HAdV-5 virions were added to the apical side of the insert and allowed to infect for 44 h. The multilayers were then sectioned and stained for α3, αV, β1, and HAdV-37 or HAdV-5. The multilayers expressed all of these proteins in the top two or three cell layers (nearest the air interface) (Fig. 7). The overall expression pattern in the multilayer did not fully correspond to the pattern of human cornea, since there was, in general, more apical expression in the multilayer. However, HAdV infection was restricted to cell layers that expressed relevant integrins, which further shows the importance of the α3, αV, and β1 subunits for entry of HAdV-37 and HAdV-5. We also noted that the β1 MAb stained the substratum interface. Taken together, we conclude that α3β1 and αVβ1 integrins are required for efficient infection of human corneal epithelial cells by HAdV-37 virions.

## DISCUSSION

Here, we found that HAdV-37 uses β1 integrin subunits in dimers with α3 and αV to infect HCE cells but not β3 and β5 integrin subunits, which are absent or close to absent from these cells. Previous work had shown that HAdV-37 interacts physically with integrin αVβ5 *in vitro* (23), but the relevance of this interaction for infection of corneal cells has not been investigated. We have found that the β3 and β5 integrin subunits are absent or are expressed in very small amounts on corneal epithelial cells and primary tissues, supporting that HAdV-37 uses other integrin heterodimers for infection of these cells. Antibodies specific for α3, αV, and β1 inhibited HAdV-37 infection of corneal cells. HAdV-37 virions also colocalized with α3 and αV subunits in corneal epithelial monolayers, and HAdV-37 showed preference for infection of cells in a multilayer model of corneal epithelium that was positive for expression of subunits α3, αV, and β1.

To identify a good model cell line for studies of integrin function in the life cycle of EKC-causing adenoviruses, we first investigated whether integrin expression on the human corneal epithelial (HCE) cell line matched the expression in human corneal tissue. The expression levels matched to a high degree, thus supporting the use of HCE for subsequent studies. Since β3 and β5 subunits in particular are of importance for many other HAdVs (23), we noted that this integrin was absent from corneal cells and hypothesized that EKC-causing HAdVs use other integrins for infection of these cells. Immunohistochemical staining of sections of corneal tissue showed that most integrin subunits were expressed at high levels on basal cells and with a gradual reduction in staining toward the squamous superficial cell layer. However, we did not observe any β5 staining at all, and only weak staining of β3 was observed in the basal cells. Instead, we observed pronounced expression of β4 (in basal cells mainly) and of β1 (in all layers). Similarly, we observed a strong expression of subunits α2, α3, α6, and αV in basal cells, decreasing toward the superficial cells. No staining or unspecific staining was observed only for α4 and α5. The presence of these integrins (α2, α3, α6, αV, β1, and β4) was consistent with previous proteomics-based approaches to corneal protein content (27). Expression of αVβ5 has been found in human corneal tissue when an αVβ5 integrin-specific antibody (clone P1F6) was used at high concentration (30). In this case the expression patterns of the αV subunit-specific antibody and the αVβ5 integrin-specific antibody were very similar. Since no β5-specific antibody was used to confirm the specific expression of β5 integrins, it could be that the expression patterns found were of αV instead of αVβ5. However, αVβ5 integrin may also be expressed in human corneal limbal cells (31), indicating that HAdV-37 also uses this integrin for infection of this subset of corneal cells. Furthermore, HAdV-37 is likely to replicate in conjunctival tissue also, where the integrins expressed are not yet known. Thus, it may be that HAdV-37 uses other integrin heterodimers for infection of these cells.

To investigate the effect of integrins on infection of corneal cells by HAdV-37, we pretreated HCE cells with integrin-specific monoclonal and polyclonal antibodies before infection. HAdV-5 uses multiple integrin heterodimers as coreceptors, including α3β1, αVβ1, αVβ3, and αVβ5 in various cellular contexts (16, 17, 23), so it was used here as a reference virus. The antibodies specific for subunits α3, αV, and β1 inhibited HAdV-37 infection, but HAdV5 infection was inhibited only by antibodies raised against αV. Assuming that the majority of α3 and αV subunits form dimers with β1 on corneal epithelial cells, these results suggest that HAdV-37 (but not HAdV-5) can use integrin α3β1 for infection of HCE cells. These results also indicate that the mechanisms of interaction of HAdV-5 and HAdV-37 with αV-containing integrins are different, since antibodies to the β1 subunit affected HAdV-37 infection but not HAdV-5 infection. It has been proposed by others that the ability of these two viruses to interact with integrins is affected by the length and flexibility of the fiber proteins (32). HAdV-37 fibers are rigid and short, whereas HAdV-5 fibers are more flexible but also much longer. Another feature of possible relevance is that the RGD-containing loop of the HAdV-37 penton base is much shorter than the corresponding loop of HAdV-5 (33, 34).

To investigate the impact of the RGD-containing loop in the HAdV-37 penton base, we analyzed the effect of pretreating HCE cells with soluble peptides that mimic the RGD-containing loop of the HAdV-37 penton base on HAdV-5 and HAdV-37 infection. Here, the proposed mechanism of inhibition would be that the RGD-containing peptides compete with virions for binding to RGD interaction sites on integrins and thereby inhibit infection. As expected, this peptide inhibited HAdV-37 infection more efficiently (65%) than HAdV-5 infection (40%). Two control peptides were used: HAdV-37 penton base with the RGD motif replaced with an AAA motif and RGD-containing hexapeptides used previously to study the function of penton base-integrin interactions (12). The AAA-containing peptide inhibited infection by both viruses weakly (by about 20%), and the hexapeptide inhibited infection by both viruses by about 50%. Thus, neither of the control peptides had any virus-specific effects. None of the peptides inhibited binding of HAdV-37 or HAdV-5 to HCE cells. We speculate here that fiber length and flexibility in conjunction with the length and structure of the penton base RGD-containing loops determine how HAdVs use integrins on corneal cells.

Another approach to studying the importance of RGD-interacting integrins as HAdV coreceptors is to use soluble, RGD-containing extracellular matrix (ECM) proteins in competition binding and infection analyses. Vitronectin, fibronectin, and laminin 511, for example, promote cell adhesion, spreading, and migration through interaction with integrins (31, 35–38). Preincubation of HCE cells with either vitronectin or laminin 511 inhibited HAdV-37 infection by 50%, thus providing additional support for the suggestion that interactions between the HAdV-37 penton base and RGD-binding integrins are necessary for efficient infection of corneal cells. Surprisingly, preincubation with fibronectin increased HAdV-37 infection almost 3-fold. We speculate that this effect is due to sialic acid-containing fibronectin being able to interact not only with host cell integrins but also with the sialic acid-binding fiber, thus serving as a bridge between virus and cell. Nevertheless, these results provide additional support for the suggestion that HAdV-37 interacts with integrins through its RGD motif. None of the ECM proteins affected the binding of HAdV-5 and HAdV-37, which is in accordance with previous results suggesting that integrins are mediators of entry and endosomal release but are not involved in attachment (12, 13). Also, none of the ECM proteins affected infection by HAdV-5, thus supporting the idea that these two serotypes have evolved to use different integrins and/or different mechanisms of interaction with certain integrins for infection of human cells. We also attempted short interfering RNA-mediated reduction of integrin expression in HCE cells. We did not observe any consistent difference in infectivity between specific siRNAs and control siRNAs, which was likely due to the insufficient knockdown of integrins (at best 80%) and the ability of these viruses to use more than one specific integrin.

Previous studies have shown that some 30% of all HAdV-2 virions are internalized within 15 min (15), which has been suggested to be mediated by interactions with integrins. Similar to this, we found in the present study that a significant amount of AF555-labeled HAdV-37 virions colocalized with α3 or αV subunits following 30 min of incubation at 37°C (Fig. 6). There is also a clear trend in that both αV and α3 integrins colocalize with HAdV-37 following incubation on ice for 15 min at 37°C. However, this observation could not be statistically validated by pixel shift analysis. This likely reflects the high abundance of integrins at the cell surface and the large cell-to-cell variability in the numbers of labeled HAdV-37 particles on cells. Taken together, these data suggest that most virions are either localized at the plasma membrane, and thereby in this case the entry process is relatively slow, or that virions and integrins are internalized together within the same compartment for up to 30 min at 37°C.

Further studies of interactions of EKC-causing adenoviruses with their cellular receptors and coreceptors have been hampered by the lack of suitable animal models. Until now, HCE cells have been used mainly as monolayers to address these questions. Here, we developed a multilayer system with these cells to study integrin function in HAdV infection. After a total of 13 days of culture in an air-liquid interface, 7 to 10 layers formed, which is similar to the five to seven layers seen *in vivo* (29). The overall organization of the multilayer tissue resembled that of human cornea, with basal-like cells in the lower and intermediate layers and squamous-like cells in the most superficial layer. In this model, all integrin-specific antibodies (to α3, αV, and β1) stained the superficial layers, which corresponded well to the staining of HAdV-5 and HAdV-37 hexon. We also noted that the β1-specific MAb also stained the substratum interface, suggesting that β1-containing integrins also localize to the basal layer of the most basal cell layer, but we cannot exclude that this MAb also binds to the artificial membrane. Thus, these studies provide novel insights into the mechanisms of corneal infection by EKC-causing HAdVs, and they may pave the way for mechanistic studies of other ocular pathogens.

## MATERIALS AND METHODS

### Cells, viruses, antibodies, proteins, peptides, and corneal tissues.

Human corneal epithelial (HCE) cells, human respiratory A549 cells, and human T-lymphoid MOLT-16 cells were grown as previously described (39–41). Species C HAdV-5 (strain Ad75) and species D HAdV-37 (strain 1477) virions were propagated with or without ^35^S labeling in A549 cells, as described elsewhere (42). The integrin-specific antibodies used for flow cytometry, *in situ* immunohistochemistry, colocalization, binding, and infection competition analyses were the following: MAbs anti-α2 (clone P1E6 [mouse]), anti-α3 (clones P1B5 [mouse] and ASC-1 [mouse]), anti-α4 (clones P4C2 [mouse] and PS/2 [rat]), and anti-α5 (clone P1D6 [mouse]), all from Merck Millipore; anti-α6 MAbs (clones GoH3 [rat] and MP 4F10 [mouse]) from Abcam; MAb anti-αV (clone 272-17E6 [mouse]) from Thermo Fisher Scientific; MAb anti-β1 (clone P5D2 [mouse]) and PAb anti-β1 (AF1778 [goat]), both from R&D Systems; MAb anti-β3 (clone MHF4 [mouse]) from Abnova; MAb anti-β4 (clone 422325 [mouse]) from R&D Systems; and PAb anti-β5 (H00003693D01P [rabbit]) from Abnova. We also used MAb anti-adenovirus hexon (clone B025/AD51 [mouse] from Abcam; for staining of HAdV-infected, artificial cornea) and MAb anti-GD1a (clone EM9 [mouse]; a kind gift from Hugh Willison; for competition binding and infection experiments). The peptides (from GenScript) used in binding and infection competition analyses were 37-RGD (NDAVPRGDNYASAAEA), 37-AAA (NDAVPAAANYASAAEA), and GRGDSP. The integrin-recognizing ligands used in binding and infection competition analyses were vitronectin (R&D Systems), fibronectin (Roche), and laminin 511 (Biolamina). Human corneas were collected from two donors at autopsy and from three patients undergoing surgery (evisceration, *n* = 2; corneal transplantation, *n* = 1), with approval from the Regional Ethical Review Board in Umeå (Dnr 2010-373-31M).

### Immunohistochemistry.

Human corneas were mounted with OCT cryomount (HistoLab Products) on cardboard and quickly frozen in propane prechilled using liquid nitrogen. The samples were stored at −80°C until further investigation. Five- to seven-micrometer sections were cut at −23°C using a Leica CM3050 cryostat (Leica Biosystems), collected on Superfrost Plus slides (Thermo Fisher Scientific), and stored at −23°C until further processing for immunohistochemistry.

The sections were processed for indirect immunofluorescence using primary antibodies to integrin subunits α2, α3 α4, α5, α6, αV, β1, β3, β4, and β5 (see above for the origins of antibodies and the legend to Fig. 1). As secondary antibodies, we used goat anti-mouse Alexa Fluor 488 (AF488), goat anti-rabbit AF488 (both from Thermo Fisher Scientific), or donkey anti-rat AF488 (Jackson ImmunoResearch).

Briefly, the sections were brought to room temperature (RT) to dry for at least 15 min, fixed with 2% paraformaldehyde (PFA) for 8 to 10 min, and washed three times in 0.1 M phosphate-buffered saline (PBS) for 15 min. All incubation steps were performed in a humidity chamber to keep the sections from drying. Before incubation overnight at 4°C with primary antibody and incubation with secondary antibody (30 min at 37°C), the sections were incubated with 5% normal serum (goat or donkey) for 15 min at RT to block nonspecific binding of secondary antibody. Finally, the sections were mounted using Vectashield mounting medium with DAPI (4′,6-diamidino-2-phenylindole; Vector Laboratories) for visualization of nuclei. Secondary antibody control sections were treated as described above, except that the primary antibodies were omitted. Labeled sections were evaluated using a Nikon Eclipse E800 microscope (Nikon) equipped with a SPOT RT KE slider camera (Diagnostic Instruments) or a Leica DM6000 B microscope with a Leica DFC 360 FX camera (Leica Microsystems). Digital images were further processed with Adobe Photoshop CS6 software (Adobe Systems).

### Infection experiments.

Adherent monolayers of HCE cells (10^5^ cells/well in a 48-well plate) were pretreated with integrin-specific antibodies diluted 1:100 (see above and the legend to Fig. 3 for details about antibodies used), peptides (4 mM), or integrin-binding ligands (vitronectin, 1 μM; fibronectin, 1 μM; laminin 511, 0.12 μM) for 1 h on ice. The cells were then washed once prior to incubation with HAdV-5 (5 × 10^7^ virions/well) or HAdV-37 (3 × 10^7^ virions/well) on ice for an additional hour. The relative numbers of HAdV-5 and HAdV-37 were not adapted with respect to the tropism of these viruses but rather in order to account for the different readouts (automated versus manual) and to account for batch-to-batch differences of infectious particles. Unbound virions were washed away with medium containing 1% fetal bovine serum (FBS) and then incubated at 37°C in the same medium. Forty-four hours later, the cells were washed once in PBS, fixed with methanol (−20°C) for 10 min, and stained with homotypic rabbit sera produced in-house (HAdV-5) or by Agrisera AB (HAdV-37), diluted 1:100 in PBS for 1 h at RT. The wells were then washed twice with PBS and incubated for an additional hour at RT with a fluorescein isothiocyanate (FITC)-conjugated swine anti-rabbit antibody (Dako) diluted 1:100 in PBS. Infection was analyzed by counting the number of infected cells (10 view fields/well) using a fluorescence microscope (Zeiss Axiovert 25; Zeiss). Experiments with RGD-containing/lacking peptides and RGD-containing ligands were performed as described above but with the following modifications: 2 × 10^4^ HCE cells were seeded on a 96-well plate, 2.3 × 10^6^ HAdV-5 virions were used, and the number of infected cells was analyzed and counted with the Trophos system (Luminy Biotech Enterprises). This modification was performed in order to adapt the assay for high-throughput imaging and to account for batch-to-batch differences of infectious particles.

### Binding experiments.

HCE cells were detached with PBS containing 0.05% EDTA, washed, and incubated in suspension for 1 h at 37°C in cell culture medium with 10% FBS before being transferred to 96-well plates (2 × 10^5^ cells/well). HCE cells were washed three times with serum-free medium before being pretreated with integrin-specific antibodies (diluted 1:100) (see above and the legend to Fig. 3 for details about antibodies used). Unfortunately, the concentration was not provided from the manufacturer for all antibodies. However, for the ones where data are available, the antibody was provided at a concentration of 1 mg/ml. All of the peptides were used at a concentration of 4 mM. Integrin-binding ligands vitronectin and fibronectin were used at 1 μM concentration and laminin 511 at 0.12 μM. Antibodies, peptides, and integrin ligands all where diluted in serum-free medium prior to binding for 1 h on ice. After washing, ^35^S-labeled virions were added (2 × 10^9^ virions/well) and incubated on ice for another hour. The cells were finally washed with serum-free medium before quantification of radioactivity using a Wallac 1409 liquid scintillation counter (Perkin-Elmer).

### Flow cytometry.

HCE, A549, and MOLT-16 cells were detached using PBS containing 0.05% EDTA and incubated in cell culture medium for 1 h at 37°C. The cells were transferred to 96-well plates (2 × 10^5^ cells/well) and incubated for 1 h on ice in fluorescence-activated cell sorting (FACS) buffer (PBS, 2% FBS, and 0.001% NaN_3_) with monoclonal or polyclonal antibodies (diluted 1:100) that specifically recognized integrin subunit α2, α3, α4, α5, α6, αV, β1, β3, β4, or β5 (see above and the legend to Fig. 2 for details about antibodies used). After unbound antibodies were washed away, the cells were incubated on ice for 1 h with FITC-conjugated anti-mouse, anti-rabbit, anti-rat, or anti-goat (all from Dako) antibody diluted 1:100 in FACS buffer. Integrin expression levels were quantified using an LRS II instrument (Becton Dickinson).

### Single-cell colocalization analysis.

CsCl-purified HAdV-37 virions stored at −80°C were first centrifuged for 5 min at 14,000 rpm to remove aggregated virions. Virions (200 μg) were then mixed with 12 μg of Alexa Fluor 555 (AF555; Thermo Fisher Scientific), and the final volume was adjusted to 800 μl in PBS, pH 7.4. The mixture was then incubated for 1 h at RT in the dark with slow agitation before being heavily vortexed and dialyzed against PBS in a Slide-A-Lyzer dialysis cassette (10,000 molecular weight cutoff [MWCO]; 0.5 to 3 ml; Thermo Fisher Scientific) overnight at 4°C. AF555-labeled HAdV-37 virions were stored in PBS containing 20% glycerol. HCE cells (3 × 10^4^) were allowed to attach to cover slides overnight at 37°C. They were then washed twice with serum-free medium. Cell samples from time point zero were washed twice with ice-cold serum-free medium (to prevent virus entry), and cell samples from the 15-min and 30-min time points were washed twice with preheated medium (37°C). AF555-labeled HAdV-37 virions were added (5.6 μg/cover slide) and incubated on ice for 30 min (time point zero) or at 37°C (15-min or 30-min samples). After viral binding and entry, the cells were washed three times with ice-cold PBS and fixed for 15 min with 4% PFA. After fixation, the cells were washed four times with PBS and blocked with 5% goat serum in 0.05% saponin–PBS for 15 min at RT. Antibodies targeting α3 (diluted 1:3,000) or αV (diluted 1:1,000) (see above and the legend to Fig. 6 for details about antibodies used) were added to colocalization buffer (CB; 1% goat serum, 0.05% saponin, and PBS), incubated at RT for 1 h, and then washed four times with CB before being incubated with AF488-conjugated goat anti-mouse antibody (1:300; Thermo Fisher Scientific) in CB for 1 h at RT. After the slides were washed four times, colocalization was analyzed by confocal microscopy using a Nikon A1R laser scanning confocal microscope with a 60× oil immersion lens under the control of NIS-Elements microscope imaging software. Images were thresholded to remove unspecific signal, and virion colocalization with anti-integrin antibodies was scored by comparing the number of fluorescent virions that colocalized with anti-integrin antibodies with the total number of cell-associated fluorescent virions. For each condition, at least 5 z-stacks containing a minimum of 2 cells or more were analyzed. The colocalization analysis was performed using NIS-Elements software, and colocalization was scored only when fluorescent virus structures colocalized in the *x*, *y*, and *z* directions with integrin antibodies. Pixel shift analysis was performed to control that the observed colocalization between virions and integrins was not due to chance. All pixels in the channel containing AF555-labeled HAdV-37 virions were shifted 1 μm in *x* and *y* and 0.5 μm in *z* compared to the integrin channel in all images. Colocalization was measured as in the original images, and the results were compared to the original data using a paired *t* test.

### Colocalization analysis of artificial corneal three-dimensional epithelium.

HCE cells in solution were counted, and 1.5 × 10^5^ viable cells were seeded in 12-well plates on transwell inserts with a diameter of 12 mm and a pore size of 0.4 μm (Corning) using growth medium supplemented with 5% FBS, 20 mM HEPES, 1× penicillin-streptomycin, and 0.1 μg/ml cholera toxin. After 3 days in culture with medium covering both the apical and the basal sides of the cells, the transepithelial resistance (TER) was measured to ensure polarization. When the TER had increased at least 5-fold, the medium was removed on the apical side, bringing the cells to an air-liquid interface (ALI). Every second day for the next 10 days, the medium was replaced on the basal side, with the apical side being washed gently with warm PBS. After 10 days of growth in ALI, the cells were infected apically with HAdV-37 or HAdV-5 using 2 × 10^5^ virus particles per insert. The infection was performed in 100 μl of DMEM (per insert) for 60 min at 37°C with gentle rocking, followed by a gentle wash with warm PBS. The infection was terminated after 48 h by fixation in ice-cold acetone overnight. The following day, the fixed inserts were cut in half and processed in glycol methacrylate as previously described (43). Sections 2 μm thick were cut and stained for adenovirus hexon and integrins (see above and the legend to Fig. 7 for details about antibodies used). Nonspecific antibody binding was blocked with 1% BSA in Tris-buffered saline, pH 8.0, for 30 min at RT and then incubated overnight with primary antibodies (dilution, 1:50). After washing away unbound primary antibodies, AF-conjugated secondary antibodies (diluted 1:1,000) directed against the primary antibody host species were applied and the slides were incubated for 2 h. All antibodies were diluted in Tris-buffered saline, pH 8.0, and added at RT. After a final wash, the slides were mounted using DAPI containing mounting medium (Vector Laboratories) and imaged using a Nikon Eclipse Ti microscope with NIS-Elements software.

### Statistical analyses.

All experiments were performed three times (*n* = 3), in duplicates or triplicates, except single-cell colocalization studies and immunohistochemistry experiments, which were performed once (*n* = 1) and twice (*n* = 2), respectively. The results are expressed as means ± standard deviations and were analyzed either by *t* test or by two-way analysis of variance with Dunnett's posttest. We used GraphPad Prism software (version 6 for Mac; GraphPad Software). All *P* values of <0.05 were considered statistically significant.
